# Electrocardiographic activity depends on the relative position between intimate persons

**DOI:** 10.1038/s41598-024-54439-5

**Published:** 2024-02-21

**Authors:** Kae Mukai, Tomoko Isomura, Ryoji Onagawa, Katsumi Watanabe

**Affiliations:** 1 Faculty of Science and Engineering, Waseda University, Building 59, 3-4-1 Okubo, Shinjuku, Tokyo Japan; 2 Japan Society for the Promotion of Science, Tokyo, Japan; 3 Graduate School of Informatics, Nagoya University, Aichi, Japan

**Keywords:** Psychology, Human behaviour

## Abstract

Interpersonal space (IPS) refers to the area surrounding the body in which we engage in social interactions while maintaining our comfort. Numerous previous studies have reported the psychological and physiological changes associated with the proximity of two people engaged in face-to-face interaction. Currently, there is limited knowledge about how the relative position between two socially intimate individuals affects their psychological and physiological states. This research measured the subjective discomfort and electrocardiographic responses of participants when standing static at various relative positions. The highest discomfort, lowest heart rate, and highest heart rate variability (HRV; parasympathetic activity index) were observed when the friend stood in the face-to-face position. Interestingly, heart rate also decreased when the friend stood on the right side, although HRV did not change. We interpreted the results as suggesting that the presence of a familiar person elicits the electrocardiographic responses associated with an increase in parasympathetic activity.

## Introduction

Communication with others is achieved by maintaining different positions and interpersonal distances. The space surrounding the body when interacting with others is called interpersonal space (IPS)^1–5^. Having adequate IPS is important for better interactions with others^6,7^.

Studies have identified the shape of the IPS based on the feeling associated with avoidance behavior when approached by others^8,9^, such as fear or discomfort related to autonomic nervous system responses^10–18^. In terms of autonomic nervous system responses, Candini et al.^19^ showed that skin conductance responses were higher when others entered the IPS. Other research has reported increased heart rate and decreased HRV when others approach the IPS^20^. Because fear is often associated with increased heart rate, decreased heart rate variability (HRV), and increased skin conductance^21,22^, these physiological changes may be caused by sympathetic activity^23,24^ in response to potentially threatening situations.

In many previous studies, the IPS was examined in a stopping distance task in which a stranger approached or retreated^19,20,25^. However, the social relationship could influence psychological and physiological responses. For example, when considering a real-life situation in which acquaintances or friends are standing next to us, the threat level might be relatively low compared to when strangers are standing next to us. Given that there is evidence that smaller IPSs may be formed for more intimate others, such as family and friends^26–28^, psychological and physiological responses may differ.

Furthermore, due to task limitations, such as the visual information-based measurement of approaching and retreating others, previous studies have often only examined interpersonal distance in front of participants. There is little research on the shape of the IPS around the body, and the findings are inconclusive^8,25,29^. For instance, Hayduk^8^ found that the anterior IPS was larger than the posterior IPS, whereas Hecht et al.^25^ observed that the shape of the personal space was similar to a circular zone. Only a few studies have compared more than two directions.

In this study, we investigated changes in psychological and physiological responses to the presence of another person at various positions in the transverse plane. Instead of the stop distance task commonly used in previous studies on the IPS, we used a static standing task with a lower level of fear. The advantage of our task is that it allows the IPS to be examined in various directions. Participants maintained a static standing posture in various relative positions (8 position conditions) with another person. Heart rate and HRV were assessed by recording participants’ electrocardiogram (ECG) data. We also assessed the subjective report of discomfort for each trial.

## Results

To test the psychological and physiological responses to the presence of another person at various positions, subjective discomfort using the visual analog scale (VAS) and R-R intervals (RRI) and HRV calculated from ECG time-series data were collected, see “Methods” section for details. The means without normalization for each condition for all variables (discomfort, RRI, and RMSSD) are shown in Table 1. The results of the two-way mixed ANOVAs for all variables are shown in Table 2. Statistical results of the original scores for all variables are presented in the Supplementary materials.

**Table 1:** Mean (SD) values without normalization of discomfort, RRI, and RMSSD in each condition.

Conditions	F-see	R-see	L-see	B-see	R-seen	L-seen	B-seen	Baseline
Discomfort								
Person	4.35 (1.33)	5.37 (0.98)	5.52 (1.19)	5.43 (1.02)	5.51 (1.26)	5.85 (1.13)	5.93 (1.26)	6.49 (1.30)
Object	4.75 (1.36)	5.39 (1.24)	5.53 (1.37)	5.48 (1.19)	5.49 (1.16)	5.41 (1.44)	5.78 (1.45)	6.01 (1.34)
RRI [ms]								
Person	747.90 (92.87)	742.89 (96.38)	727.83 (94.22)	723.91 (93.77)	734.00 (100.07)	725.01 (92.61)	714.38 (93.55)	710.75 (92.39)
Object	732.94 (98.98)	728.82 (102.16)	734.70 (109.55)	744.03 (123.80)	722.39 (100.18)	729.85 (108.19)	739.15 (112.03)	741.28 (119.95)
RMSSD [ms]								
Person	27.66 (11.30)	20.76 (9.61)	20.74 (8.28)	21.18 (10.07)	22.15 (9.50)	21.81 (8.63)	21.57 (8.81)	20.99 (8.89)
Object	20.97 (12.79)	19.75 (11.86)	19.72 (11.02)	22.41 (12.30)	20.66 (12.06)	20.98 (12.35)	20.25 (12.63)	20.48 (12.29)

**Table 2:** Statistical results of two-way mixed ANOVAs on discomfort, RRI, and RMSSD.

Variables	Effect factor	F	df	p	η_p_^2^
Discomfort	Condition	13.117	7, 308	< 0.001	0.230
	Condition * Group	1.074	7, 308	0.380	0.024
	Group	1.861	1, 44	0.179	0.041
RRI	Condition	3.035	7, 294	0.004	0.067
	Condition * Group	6.759	7, 294	< 0.001	0.139
	Group	11.962	1, 42	0.001	0.222
RMSSD	Condition	9.420	7, 294	< 0.001	0.183
	Condition * Group	7.739	7, 294	< 0.001	0.156
	Group	1.763	1, 42	0.191	0.040

### Subjective report of discomfort

**Figure 1:**
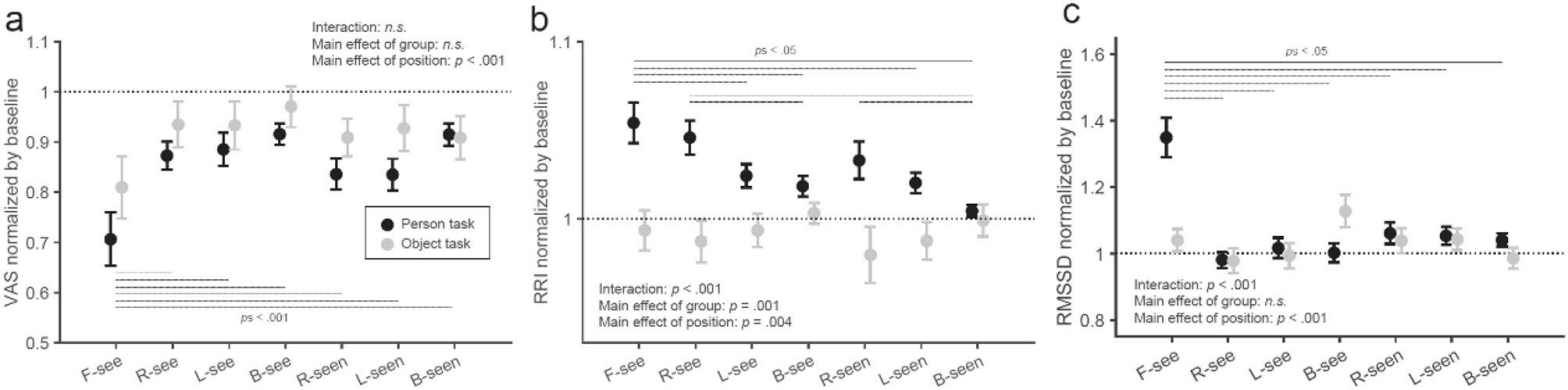
Plots of discomfort, RRI, and RMSSD ratio to the baseline condition. Error bars indicate the standard errors.

Figure 1a shows the ratio of the average discomfort scores for each condition to the baseline (back-to-back) condition. Lower scores indicate greater discomfort. A two-way mixed ANOVA revealed a significant main effect of the position condition (*F*_[7, 308]_ = 13.117, *p* < 0.001, η_p_^2^ = 0.230). A simple main effect test indicated that a significantly lower discomfort score (i.e., higher discomfort feeling) was observed in the F-see condition than those in all other conditions (*p*_Holm_s < 0.001). The discomfort scores in the R-see, L-see, R-seen, L-seen, and B-seen conditions were significantly lower than that in the baseline condition (*p*_Holm_s < 0.05). The main effect of the group (*F*_[1, 44]_ = 1.861, *p* = 0.179, η_p_^2^ = 0.041) and interaction (*F*_[7, 308]_ = 1.074, *p* = 0.380, η_p_^2^ = 0.024) were not significant.

### Heart rate

#### R-R intervals (RRI)

Figure 1b shows the average RRI scores for each condition to the baseline (back-to-back) condition. A two-way mixed ANOVA revealed a significant interaction (*F*_[7, 294]_ = 6.759, *p* < 0.001, η_p_^2^ = 0.139). Post-hoc analysis revealed that the RRI ratio of the F-see condition in the Person task was significantly higher than that of all conditions except those of the R-see and R-seen conditions in the Person task and those of all conditions in the Object task (*p*_Holm_s < 0.05). The RRI ratio of the R-see condition in the Person task was significantly higher than those of the B-see, B-seen, and Baseline conditions in the Person task and those of all conditions in the Object task (*p*_Holm_s < 0.05). The RRI ratio of the R-seen condition in the Person task was significantly higher than that of B-seen and Baseline conditions in the Person task and those of R-see, R-seen, and L-seen conditions in the Object task (*p*_Holm_s < 0.05). The main effect of the position condition (*F*_[7, 294]_ = 3.035, *p* = 0.004, η_p_^2^ = 0.067) and that of the group (*F*_[1, 42]_ = 11.962, *p* = 0.001, η_p_^2^ = 0.222) were significant.

#### Root mean square of successive RRI differences (RMSSD)

Figure 1c shows the average RMSSD scores for each condition to the baseline (back-to-back) condition. A two-way mixed ANOVA revealed significant interactions (*F*_[7, 294]_ = 7.739, *p* < 0.001, η_p_^2^ = 0.156). Post-hoc analyses indicated that RMSSD of the F-see condition in the Person task were significantly greater than those of all other conditions (*p*_Holm_s < 0.001). The main effect of the position condition was significant (*F*_[7, 294]_ = 9.420, *p* < 0.001, η_p_^2^ = 0.183). The main effect of the group was not significant (*F*_[1, 42]_ = 1.763, *p* = 0.191, η_p_^2^ = 0.040).

## Discussion

The results showed that the greatest discomfort, the greatest decrease in heart rate, and the greatest increase in HRV occurred when others were standing in front. In addition, the decrease in heart rate was observed when a person was on the right side. The changes in electrocardiographic responses occurred when a person, not an object, was present.

We found that heart rate decreased when another person was present in specific relative positions (front and right). In addition, HRV tended to increase, i.e., parasympathetic activity increased when standing facing another person. This result could be raised by the social relationship and social situation in the current experimental setting. Given the evidence that the presence of a romantic partner or spouse in a good relationship activates parasympathetic activity, such as low resting blood pressure and higher HF-HRV^30,31^, the observed decrease in heart rate may have been due to increased parasympathetic activity during interactions with others who were not as close as romantic partners and spouse but still had relatively close relationship with them. It has been suggested that invasion of IPS induces negative emotions associated with avoidance behaviors such as fear and discomfort^8,9^ and an increased skin conductance response and decreased heart rate variability was observed^19,20^. In the social situation with an approaching stranger used in many previous studies, people may experience discomfort associated with increased sympathetic responses. Candini et al.^19^ reported that an increase in skin conductance response was not observed in social situations with a relatively low degree of threat, such as a stranger moving away (withdrawal condition). In the current study, two people were standing stationary, which may have been a low threat level situation compared to approaching situation, as in the withdrawal condition. It is possible that activities of automatic nervous system are sensitive to the social context of social intimacy or the type of situation in which they are interacting.

Interestingly, a decrease in heart rate was also observed when another person was on the right side. One possible explanation for this asymmetry could be the influence of handedness. Gérin-Lajoie et al.^32^ reported that the physical distance between a person and an object was shorter on the dominant side of the hand than on the non-dominant side when bypassing an object. Since all but one of the participants in this study was also right-handed, the IPS on the right side might be smaller than on the left side. This asymmetry in IPS might have produced differences in the electrocardiographic responses received when standing with others. However, since the increased parasympathetic activity was not observed when others were on the right side, there might be a different mechanism of the autonomic activity underlying the right-side specific electrocardiographic response than when others were in the front. To explore this possibility, autonomic activity would need to be examined in terms of multiple physiological indices. Our study could not quantify sympathetic activity because the data length of this study was not sufficient to estimate sympathetic activity. Future studies should investigate this possibility by simultaneously measuring sympathetic activity, which can be examined from electrodermal response and long-term ECG data, indices to follow the relationship between the IPS and the autonomic nervous system. Furthermore, considering that the activity of the autonomic nervous system is influenced by body movements^33,34^, the possibility of body movements depending on the relative position conditions could not be ruled out. Simultaneous measurement of body movements would help to clarify whether the increase in parasympathetic activity in a particular relative position is due to body movement or not. In addition, the back-to-back condition was set as the baseline condition in both the person and object tasks to normalize the data in the current study. Given that potential differences between the baseline conditions for both tasks could not be eliminated due to the differences in what was behind the two tasks, it is necessary to measure the resting autonomic nervous system and set it as the baseline condition in future studies.

Although we measured the changes in electrocardiographic responses at only one interpersonal distance in the current study, it has been suggested that individual differences exist in comfortable spaces with others, and that psychological and physiological responses may differ even when another person is physically present at the same distance^19^. One possible individual characteristic behind these individual differences in IPS is interoceptive accuracy^35,36^. Ferri et al.^35^ reported that individuals with high and low interoceptive accuracy differ in their autonomic responses to the presence of others around their peripersonal space. Although the relationship between peripersonal space and IPS is still on the table^37^, if both have the meaning of boundaries that include the self and others, it is possible that there is an association between personal characteristics such as interoceptive accuracy and the IPS.

Our findings suggest that electrocardiographic responses elicited by the invasion of the IPS by others have the potential to define the boundaries between self and others. We also believe that our reports that the physical reactions can vary in social contexts provide a new perspective on a number of studies of IPS that have primarily examined explicitly psychological variables.

## Conclusion

This study showed that increased discomfort, decreased heart rate, and increased HRV occurred when others with high social closeness were present in front. These results may have been caused by the presence of a familiar person increasing the parasympathetic activity. Although a previous study reported that the approach of a stranger elicits sympathetic activity, the present study suggests that the physiological responses elicited differ depending on the social context.

## Methods

Before conduction the experiment, the study was approved by the Ethics Committee of Nagoya University (No. NUPSY-210901-L-01) and all study methods were performed in accordance with the relevant guidelines and regulations approved by the Research Ethics Committee. Informed consent was obtained from all participants prior to their participation in this study.

### Experimental task

Two tasks were used to investigate the effect of the presence of another person on one’s psychological and physiological state. One was a Person task in which participants were paired with a human partner. The other was an Object task in which an object was present in the surroundings instead of a human partner.

#### Person task

**Figure 2:**
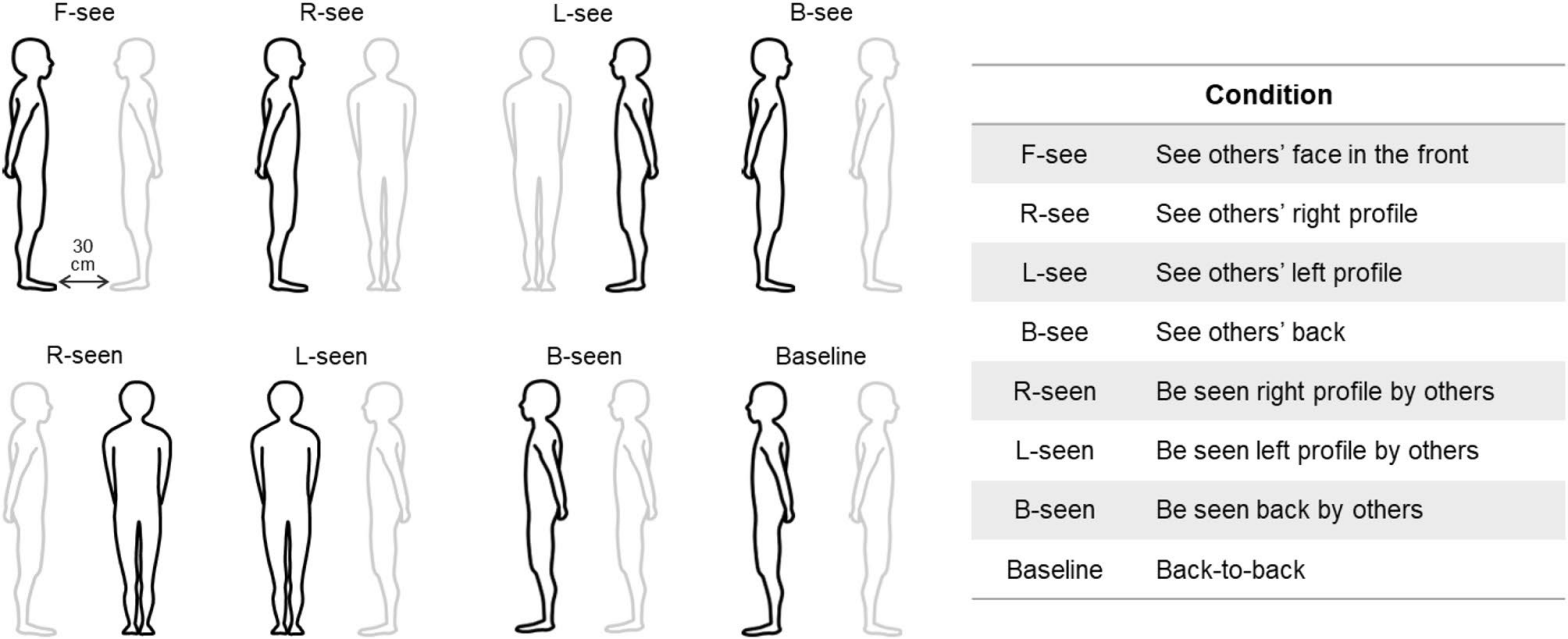
Experimental conditions for the Person task. Paired participants maintained a static standing posture with their hands crossed behind their backs for 60 s. Eight conditions were set regarding the positional relationship between the two paired participants.

Two paired participants (friend pair) maintained a static standing posture for 60 s. They crossed their arms behind their backs to reduce fatigue from prolonged standing posture. Eight conditions were set regarding the positional relationship between the two participants (Fig. 2), as follows. In the F-see condition, participants were required to maintain a static standing posture face-to-face with friends, with their eyes fixed on the center of the partner’s eyes. In the L-see, R-see, and B-see conditions, they stood while looking at the friends' right profiles, left profiles, and backs with their eyes fixed on a point on the friends' heads, respectively. In the F-seen, L-seen, and B-seen conditions, they stood while friends looked at their right profiles, left profiles, and backs, respectively. In the baseline condition, they stood back-to-back with their friends. In the F-seen, L-seen, B-seen, and baseline conditions, they kept their eyes on the fixation point 30 cm in front of them. In all conditions, the distance between paired participants was 30 cm.

#### Object task

Participants maintained a static standing posture under the same conditions as in the Person task with an object placed in the same location. The object was a cylinder of the same height as each participant. The width of the object was 20 cm (circumference: 63 cm). In all conditions, participants were required to fixate a fixation point 30 cm in front of them while performing the Object task.

For both tasks, participants provided subjective ratings of comfort/discomfort using a visual analog scale (VAS) (0: lowest comfort—10: greatest comfort) after performing each trial. Participants' ECGs during the task were recorded with MP160 and Acknowledge (BIOPAC System Inc., US) at a sampling rate of 2000 Hz.

### Participants

Fifteen Japanese friend pairs (30 participants, 16 females, mean age = 20.1 ± 1.4 years, mean height = 163.5 ± 7.3 cm) participated in the Person task. Sixteen Japanese adults (15 females, mean age = 20.4 ± 1.8 years, mean height = 161.6 ± 6.5 cm) participated in the Object task. There was no overlap of participants between the two experiments. The average height difference between the two paired participants in the Person task was 4.5 cm. The dominant hand of all participants was right-handed, except for one participant in the Person task. All had normal or corrected-to-normal vision.

### Procedure

Participants were instructed to maintain a stationary standing posture for 60 s, facing the direction specified for each condition. Participants completed a total of 32 trials, including 4 trials in eight conditions. The order of trials was pseudo-randomized with a constraint that the same condition could not appear two or more times in a row. The duration of the trials was approximately one and a half hours (Person task) or one hour (Object task), including breaks.

### Data analysis

The participants’ ECG time series data were high-pass filtered at 1 Hz and low-pass filtered at 35 Hz. For analysis, we used ECG data for a length of 60 s. Using the participant's ECG data, we detected the R wave's peak values and calculated the mean R–R interval (RRI) for each condition. Errors and noise were visually inspected during the RRI detection process. The subsequent analysis included ECG data of 14 pairs (28 participants) in the Person task group and 16 participants in the Object task group, except for one pair whose ECG data was not recorded successfully.

Moreover, HRV was evaluated employing root mean square of successive RRI differences (RMSSD)^38–42^. This methods assess parasympathetic activity of the autonomic nervous system using only a small amount of data^40–42^. The RMSSD was calculated by the following formula (1):

1 $$\begin{array}{c}RMSSD = \sqrt{\frac{1}{N-1}{\sum }_{i=0}^{n}{\left({RR}_{i+1}- {RR}_{i}\right)}^{2}}\end{array}$$

RMSSD reflects rapid beat-to-beat variability and is primarily associated with parasympathetic activity^40^. RMSSD were calculated from data points for 60 s on each trial, as was RRI.

### Statistical analysis

Since there was no significant difference in baseline condition between the person and object conditions (see Supplementary materials), the ratio to the baseline condition was calculated by dividing the values of all variables (discomfort, RRI, and RMSSD) for each condition by the value of each participant’s baseline condition in order to control individual differences in psychological and electrocardiographic responses. Two-way mixed ANOVAs (group [2: Person vs. Object] × position condition [8]) were performed. All statistical analyses were performed with JASP 0.16.4.0^43^. The level of statistical significance was set at *p* < 0.05.

### Supplementary Information

Supplementary Information.

## Data Availability

Analyzed data and commented analysis script are available on the Open Science Framework (OSF): https://osf.io/txyfc/?view_only=41f08b5f6aa144a9a0e1b13f8af3200c.
