# Supplementary material for: Electrocardiographic activity depends on the relative position between intimate persons
Source: Sci Rep. 2024 Feb 21;14:4281. doi: 10.1038/s41598-024-54439-5 (PMC10882048; doi:10.1038/s41598-024-54439-5)
Supplement: Supplementary file 1 — Supplementary Information. [file 41598_2024_54439_MOESM1_ESM.pdf]

Supplementary material

Subjective reports of discomfort

Fig. 4a shows the mean discomfort rating for each condition. A two-way mixed ANOVA revealed a significant main effect of the position condition ($F_{[7, 308]} = 14.485$, $p < .001$, $\eta_p^2 = 0.111$). A simple main effect test indicated that a significantly lower discomfort score (i.e., higher discomfort feeling) was observed in the F-see condition than those in all other conditions (p_{Holm}s < .001). The discomfort scores in the R-see, L-see, R-seen, L-seen, and B-seen conditions were significantly lower than that in the baseline condition (p_{Holm}s < .05). The main effect of the group ($F_{[1, 44]} = 0.064$, $p = .802$, $\eta_p^2 < 0.001$) and interaction ($F_{[7, 308]} = 1.223$, $p = .289$, $\eta_p^2 = 0.009$) were not significant.

R-R intervals (RRI)

Fig. 4b shows the mean RRI for each condition. A two-way mixed ANOVA revealed a significant interaction ($F_{[7, 294]} = 7.959$, $p < .001$, $\eta_p^2 = 0.159$). Post-hoc analysis revealed that the non-normalized RRI of the F-see condition in the Person task was significantly higher than those of the L-see, B-see, L-seen, B-seen, and baseline conditions in the Person task (p_{Holm}s < .05). The RRI of the R-see condition in the Person task was significantly higher than those of B-seen and baseline conditions in the Person task (p_{Holm}s < .001). The RRI of the R-seen condition in the Person task was significantly higher than those of the B-seen and baseline condition in the Person task (p_{Holm}s < .05).

RMSSD

Fig 4c shows the mean RMSSD for each condition. A two-way mixed ANOVA revealed a significant interaction (RMSSD: $F_{[7, 294]} = 7.319$, $p < .001$, $\eta_p^2 = 0.148$). Post-hoc analysis indicated that the RMSSD of the F-see condition in the Person task was significantly greater than those of all other conditions in the Person task (p_{Holm}s < .001).

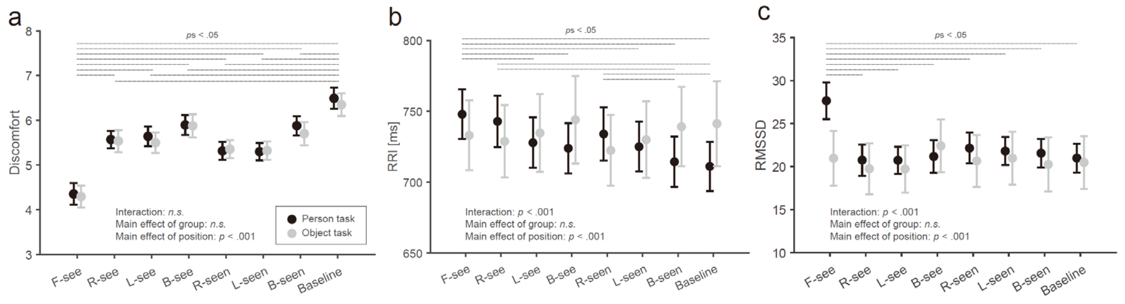

Figure 3. Plots of non-normalized discomfort, RRI, and RMSSD. Error bars indicate the standard errors.
